# Bilateral ovarian granulocytic sarcoma as the primary manifestation of acute myelogenous leukemia treated with allogenic stem cell transplantation: A case report

**DOI:** 10.1097/MD.0000000000018390

**Published:** 2019-12-27

**Authors:** Jung Yoon Choi, Hyun-Young Kim, Min Gyu Kang, Jeong Kyu Shin, Won Seop Lee, Haa-Na Song

**Affiliations:** a Division of Hemato-oncology, Department of Internal Medicine; b Department of Laboratory Medicine, Samsung Medical Center, Seoul; c Division of Cardiology, Department of Internal Medicine; d Department of Obstetrics and Gynecology, Gyeongsang National University of Medicine and Gyeongsang National University Hospital, Jinju, Korea.

**Keywords:** acute myeloid leukaemia, granulocytic sarcoma, stem cell transplantation

## Abstract

**Rationale:**

Granulocytic sarcoma (GS), also known as chloroma, is a tumor comprising myeloblasts or monoblasts, potentially occurring as an extramedullary mass. Systemic chemotherapy should be used to induce complete remission. However, such patients with chloroma have a poorer treatment outcome than those without extramedullary myeloid sarcomas.

**Patient concerns:**

A 30-year-old woman who initially presented with bilateral ovarian masses and splenomegaly was admitted to hospital. Also, her complete blood cell counts showed pancytopenia and blood smear revealed a few immature cells (3%).

**Diagnoses:**

A bone marrow biopsy demonstrated acute myelomonocytic leukemia, and the chromosomal analysis revealed a 46, XX, del18 (p11) [20] karyotype and cytogenetics and molecular markers showed all negative results.

**Interventions:**

Since this diagnosis, she received remission-inducing chemotherapy comprising anthracycline and cytarabine, which is a standard regimen for acute myeloid leukemia (AML), and followed by allogenic hematopoietic stem cell transplantation from Human leukocyte antigen (HLA)-identical sibling donor.

**Outcomes:**

After transplantation, the bone marrow engrafted successfully without complications. She visited our clinic regularly with no evidence of leukemia relapse or graft-versus host disease.

**Lessons:**

This report represents the first case of ovarian GS, wherein treatment was successful with high-dose chemotherapy, followed by allogenic hematopoietic stem cell transplantation without oophorectomy.

## 1 Introduction

Acute myelogenous leukemia (AML) is a clonal malignancy of hematopoietic tissues, characterized by accumulation of leukemic blast cells in marrow and impairment in normal hematopoiesis.

Granulocytic sarcoma (GS), also known as chloroma, is a tumor comprising myeloblasts or monoblasts, potentially occurring as an extramedullary mass.^[1]^ They may develop in any location, including the skin, bone, breast, spleen, and the peripheral and central nervous system. When myeloid sarcomas are the initial manifestation of AML, the appearance of leukemic blasts in the blood or bone marrow may occur after weeks or months. Systemic chemotherapy should be used to induce complete remission, rather than local therapy including radiotherapy. However, such patients with chloroma have a poorer treatment outcome than those without extramedullary myeloid sarcomas.^[2]^

Herein, we report the case of a 30-year-old woman with AML, who initially presented with bilateral ovarian chloroma and was treated with systemic chemotherapy, followed by successful allogenic stem cell transplantation. The Institutional Review Board of Gyeongsang National University of Hospital approved this retrospective case study and waived the requirement for informed consent.

## 2 Case report

**Figure 1:**
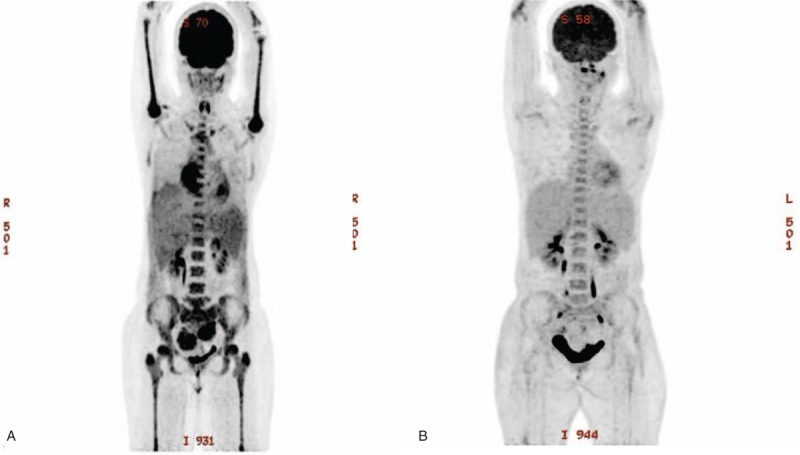
(A) Baseline positron emission tomography (PET) images showing bilateral ovarian masses with mass and nodules of pericardial invasion of the right atrium with pericardial effusion and heterogeneously increased 2-deoxy-2-fluoro-D-glucose (FDG) uptake. (B) Follow-up PET scan revealed a markedly decreased size and obliteration of FDG uptake in both ovarian masses.

A 30-year-old woman was admitted to our hospital for dry cough lasting 1 month. She presented fever and chills, and her serum C-reactive protein levels elevated to 123.3 mg/L. Furthermore, her hemoglobin level was 8.9 g/dl, which was iron-deficiency anemia. Computed tomography (CT) revealed an approximately 6.4 cm × 2 cm-sized heterogenous soft tissue masses in both adnexa with splenomegaly, mass, and nodules of pericardial invasion of the right atrium with pericardial effusion and bilateral pulmonary edema. Thus, we performed positron emission tomography (PET), which revealed 2 adnexal masses with heterogeneous focal increased 2-deoxy-2-fluoro-D-glucose (FDG) uptake (maximum standardized uptake values (SUVmax 7.2) and heterogeneously increased FDG uptake along the pericardium (SUVmax 8.8), right pleura (SUVmax 4.3), bone marrows. Furthermore, splenomegaly with heterogenous increased FDG uptake (SUVmax 4.6) was observed (Fig. 1-A).

**Figure 2:**
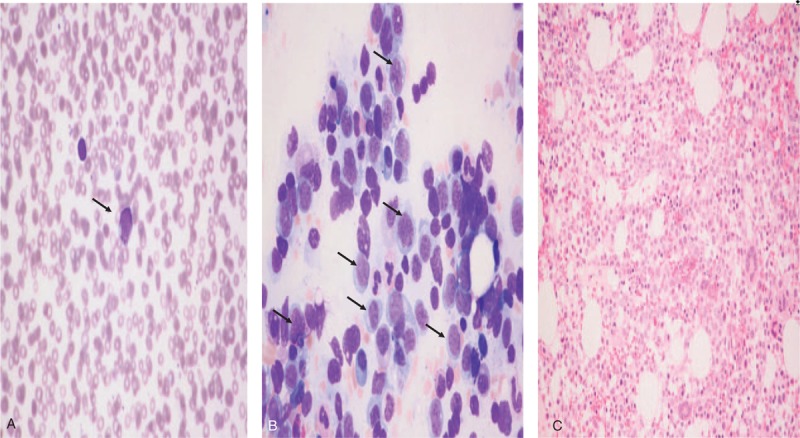
(A) A peripheral blood smear showing a few immature cells (arrow, ×400). (B) Bone marrow aspiration showing Medium to large sized myeloblasts and monoblasts are increased (Wright Giemsa stain, arrow, ×400). (C) Bone marrow biopsy showing hypercellular marrow with increased leukemic blasts diffusely infiltrated into the specimen (hematoxylin and eosin staining, ×200).

One week later, her leukocyte counts abruptly decreased to 2050/mm^3^; hemoglobin, 8.6 g/dl; platelet count, 181,000/mm^3^. Furthermore, a peripheral blood smear revealed a few immature cells (3%) (Fig. 2-A). Smears of bone marrow aspirates demonstrated increased medium to large sized blasts, including myeloblasts and monoblasts accounting for 32% of total nucleated cells and bone marrow biopsy revealed hypercellular marrow, with 90% cellularity (Fig. 2-B, C). The immunohistochemical profile of the tumor cells was as follows: positive for CD45 and myeloperoxidase (MPO) on granulocyte. Moreover, chromosomal analysis revealed a 46, XX, del18 (p11) [20] karyotype and cytogenetics examination, especially fluorescence in situ hybridization (FISH), including AML1/ETO rearrangement, PML/RARA rearrangement, CBFB/MYH11 rearrangement, MLL rearrangement, showed all negative results. The molecular markers, including a Nucleophosmin-1 (NPM1) gene mutation, CCAAT/enhancer-binding protein-a (CEBPA) gene mutation, Fms-like tyrosine kinase-3 gene(FLT3)-Internal tandem duplication (ITD) gene mutation were not detected in direct sequencing, and multiplex reverse transcriptase PCR assay (Hemavision, DNA technology, Aarhus, Denmark) showed negative result. The Hemavision panel include 28 different translocation or chromosomal rearrangements, including t(1;11)(q21;q23), t(8;21)(q22;q22), inv(16)(p13;q22), t(9,22)(q34;q11). Therefore, patient was finally diagnosed as acute myelomonocytic leukemia and she received remission-inducing chemotherapy comprising anthracycline and cytarabine, which is a standard regimen for AML. An anthracycline drug, such as daunorubicin or idarubicin, is given in a single intravenous (IV) dose on each of 3 days during the first week of treatment and cytarabine also given continuously for 7 days through an IV line. About 14 days after chemotherapy started, febrile neutropenia was developed and she received empirical antibiotics and granulocyte colony stimulating factor daily. After recovery from cytopenia, sequential bone marrow examination revealed no current residual leukemic cells. Furthermore, CT and PET follow-up examination revealed markedly decreased size and obliteration of FDG uptake of both ovarian masses, obliterated activity of heterogeneous FDG uptake along the pericardium and right pleural infiltration, along with decreased activity of splenomegaly and bone marrow (Fig. 1-B).

She received 1 cycle of post-remission chemotherapy with high dose of cytarabine twice daily at a 3 g/m^2^ dose on days 1, 3, and 5. Four weeks later, she underwent allogenic hematopoietic stem cell transplantation from Human leukocyte antigen (HLA)-identical sibling donor. Her bone marrow engrafted successfully without complications, and she visited our clinic regularly for a year with no evidence of leukemia relapse or graft-vs host disease.

## 3 Discussion

GS is a rare extramedullary tumor, usually occurring in 3% to 9% of patients with acute and chronic myeloid neoplasms.^[1]^ It comprises various degrees of myeloblasts and can originate anywhere in body; however, the involvement of female genitalia is extremely rare.

A few cases of GS in the female genitalia have been reported. Ding et al reported a small case series of a patient presenting with ovarian chloroma as the primary manifestation of AML.^[3]^ Among the 9 patients with ovarian GS, most present with abdominal pain (6/9, 66.7%). A single ovary is involved in 88% of cases, predominantly the right ovary. Bilateral ovarian masses have been reported in only 1 patient; however, that patient was diagnosed with infantile myelomonocytic leukemia and did not receive any treatment.^[4]^ Among the 9 patients with ovarian GS without leukemia, 6 (67%) patients developed AML between 5 days and 27 months (mean interval of 10.5 months) after diagnosis.^[5]^ In the present case, our patient was diagnosed with AML 1 week after presentation of bilateral ovarian GS. Furthermore, all ovarian GS patients diagnosed with AML received oophorectomy, followed by high-dose chemotherapy.[^3^,^6^,^7^,^8^,^9^,^10^,^11^] Moreover, there were no data regarding allogenic hematopoietic stem cell transplantation.

Olivia et al also reported a small case series of GS in the female genitalia, including the vagina, cervix, and ovary.^[8]^ Seven patients with ovarian GS were reported, 2 presenting with bilateral ovarian masses, and all 7 patients underwent oophorectomy, 4 received post-surgical chemotherapy, and none of them underwent hematopoietic stem cell transplantation. The prognosis is poor; in nearly all case series; however, it is suggested that patients with GS initially treated aggressively with chemotherapy may have prolonged survival without the development of acute leukemia.

We present the case of a patient with AML, who presented with ovarian GS with pleural and pericardial effusion treated via chemotherapy, followed by allogenic hematopoietic stem cell transplantation. To our knowledge, this report represents the first case of ovarian GS, wherein treatment was successful with high-dose chemotherapy, followed by allogenic hematopoietic stem cell transplantation without oophorectomy. In conclusion, we believe that allogenic hematopoietic stem cell transplantation could serve as a suitable alternative to treat AML patients presenting with ovarian GS without degrading the patient's quality of life. Further studies regarding the efficacy of stem cell transplantation for patients with ovarian GS are warranted.

## Author contributions

**Conceptualization:** Min Gyu Kang.

**Data curation:** Hyun-Young Kim, Jeong Kyu Shin.

**Formal analysis:** Haa-Na Song.

**Investigation:** Won Seop Lee.

**Resources:** Haa-Na Song.

**Writing – original draft:** Jung Yoon Choi.

**Writing – review & editing:** Haa-Na Song.

Haa-Na Song orcid: 0000-0002-7229-0153.
